# Pathophysiology of copeptin in kidney disease and hypertension

**DOI:** 10.1186/s40885-017-0068-y

**Published:** 2017-06-13

**Authors:** Baris Afsar

**Affiliations:** 1 Department of Internal Medicine, Nephrology Division, Suleyman Demirel University, Faculty of Medicine, Isparta, Turkey; 2 Suleyman Demirel University, Çünür, Doğu yerleşkesi, Isparta Merkez/Isparta, Postal Code: 32260 Isparta, Turkey

**Keywords:** Albuminuria, Copeptin, Kidney damage, Chronic kidney disease

## Abstract

Copeptin is derived from the cleavage of the precursor of arginine vasopressin (AVP), produced in an equimolar ratio in hypothalamus and processed during axonal transport AVP is an unstable peptide and has a short half-life of 5–20 min. Unlike AVP, copeptin is a stable molecule and can easily be measured. Recent evidence suggest that increased copeptin levels have been associated with worse outcomes in various clinical conditions including chronic kidney disease (CKD) and hypertension. In this review, the data regarding copeptin with kidney function (evaluated as glomerular filtration rate, increased albumin/protein excretion or both) and hypertension with regard to performed studies, prognosis and pathogenesis was summarised.

## Background

Copeptin firstly described in 1972 by Holwerda is 39-amino acids glycopeptide with leucine-rich core segment [1]. It is derived from the cleavage of the precursor of arginine vasopressin (AVP), produced in an equimolar ratio in hypothalamus and processed during axonal transport [2]. AVP is an unstable peptide, both in vivo and ex vivo, and has a short half-life of 5–20 min [3]. Unlike AVP, copeptin is a stable molecule and can easily be measured [4]. This fact stimulated the research regarding copeptin –as a measure of AVP - in various clinical conditions. Elevated levels of copeptin serve as a prognostic marker for unfavorable outcome in sepsis, shock, pneumonia, stroke, acute coronary syndrome and diabetes [5–7]. Additionally copeptin levels have been associated with kidney function in various studies.

In this review, the data regarding copeptin with kidney function (evaluated as glomerular filtration rate, increased albumin/protein excretion or both) with regard to performed studies, prognosis and pathogenesis was summarised.

Pub Med/Medline was searched for previous relevant reports. The search terms included copeptin and albuminuria, copeptin and proteinuria, copeptin and chronic kidney disease and copeptin and hypertension. When these terms are separately analyzed, they were mostly found to be duplicate. In final analysis, 22 studies were included for this review (Tables 1 and 2). These studies are heterogeneous with respect to inclusion criteria, design of the study and outcome measurement. Some studies included only chronic kidney disease (CKD) patients and some studies included healthy population and some studies included both (Table 1). Some studies examined only baseline relationships but others also investigate longitudinal data. In Tables 1 and 2 these studies are summarized with exclusion of dialysis patients.

**Table 1:** Studies Regarding Relationship of Copeptin with Glomerular Filtration Rate Albuminuria/proteinuria and Clinical Outcomes

Study	With GFR	With Albuminuria/proteinuria	Subjects	Main Finding
Meijer et al. [43]	No data	No data	548 patients with renal transplantation	-Median follow-up was 3.2 years. -Mean changes in eGFR during follow-up (3.2 yars) were -0.03, -0.44, and -1.06 mL/min/1.73 m2 per year (p:0.02) according to increasing copeptin tertiles -In multivariate regression analysis, the association of copeptin with change in eGFR remained significant after adjustment
Meijer et al. [12]	Copeptin and eGFR were negatively associated (crude β:-0.17, *P* < 0.0001	Copeptin associated with UAE (R:0.20, P:0.001).	7593 participants with baseline urinary albumin concentration >10 mg/l	With increasing quintiles of copeptin levels, microalbuminuria increased from 13–25% in males and from 8–15% in females.
Meijer et al. [44]	Copeptin and eGFR were negatively asssociated (R:- 0.58, *P* < 0.0001)	Copeptin and albuminuria were positively asssociated R:0.39, *P* < 0.0001)	102 ADPKD patients	Copeptin was positively associated with renal volume R:0.47, *P* < 0.0001) and negatively with effective renal blood flow (R: -0.52, *P* < 0.0001)
Boertien et al. [45]	Copeptin and mGFR (inulin clearance) were inversely associated (std B:-0.258, P: 0.02)	No data	79 ADPKD subjects	-Patients who started RRT had higher copeptin levels compared to subjects who did not start RRT [4.10 (3.27–17.6) versus 2.27 (1.55-5.19) pmol/L, P:0.01]
Riphagen et al. [46]	eGFR decreased from 68 ± 14, 67 ± 15, 59 ± 18, as going copptin tertile 1 to tertile 3 (*P* < 0.0001)	ACR increased from 1.6 (0.9-4.0), 1.7 (0.8-6.1) 2.7 (1.0–8.2), as going copptin tertile 1 to tertile 3 (*P* < 0.0001)	1.195 patients with T2DM	-Log copeptin was associated with CV (HR 1.17 (95% CI 0.99-1.39); P:0.068) and all cause mortality (1.22 [1.09-1.36); P:0.001) after adjustment. -However, copeptin did not substantially improve risk prediction for CV event (integrated discrimination improvement and all-cause mortality beyond currently used clinical markers.
Velho et al. [47]	Patients with highest tertile of co-peptin has lowest eGFR	Patients with highest tertile of co-peptin has Highest 24 h UAE	3.101 Type2DM patients with microalbuminuria (UAE, 20-200 mg/L) or macroalbuminuria (UAE > 200 mg/L) without renal failure at baseline	-The yearly variations of eGFR during follow-up by tertiles of plasma copeptin were 20.65 ± 0.24, 20.77 ± 0.24 and 21.91 ± 0.24 mL/min/1.73 m^2^ per year, respectively (ANCOVA P : 0.0001), adjusted for sex and age -HR for plasma copeptin tertiles as a risk for renal events (defined as doubling of serum creatinine or development of end-stage renal disease) was 4.79 (95% CI, 2.48–9.24; P, 0.0001; for T3 vs. T1). -This association remained significant when adjusted forbaseline UAE and eGFR 2.97 (1.56–6.14), P: 0.0006
Boertien et al, [48]	eGFR and baseline copeptin were negatively associated R: -0.143, *P* < 0.0001	logACR and baseline copeptin were positively associated R: 0.162, *P* < 0.0001	-1.328 patients with T2DM (349 (RAASi) and 979 without (RAASi)	-In multivarite analysis in 979 patients (without RAASi) baseline copeptin was associated with logACR; (β: 0.13, *P* < 0.001, and with eGFR, β −0.20, *P* < 0.001 -In 756 patients who were followed for 6.5 years baseline copeptin was not associated with increase in ACR after adjusment (β: 0.07, P:0.08) but associated with a decrease in eGFR ( β -0.09, P:0.03) -There was no significant association between copeptin and change in ACR or eGFR in patients using RAASi at baseline.
Boertien et al. [49]	Baseline copeptin is correlated with mGFR, R:-0.286, *P* < 0.001	No data	-241 ADPKD patients with creatinine clearance >70 mL/min	-After a 8.5 (IQR, 7.7-9.0) years follow-up copeptin was significantly associated with change in TKV after adjusting for gender, age, cardiovascular risk factors and diuretic use (*p* = 0.03). -Copeptin level was borderline significantly associated with change in mGFR after adjusting for these variables (*p* = 0.09).
Li et al. [50]	GFR and copeptin were inversely assocaited with (R:0.571, *P* < 0.001)	No data	86 non-dialysis patients with CKD and 20 control patients	-Among CKD patients, who had atherosclerotic plagues as measured by CIMT and left ventricular hypertrophy, had higher co-peptin levels compated to CKD patients without these pathologies. -Elevated co-peptin was independently associated with GFR, left ventricular hypertrophy, CIMT and previous history of CVD in multivariate analysis.
Sontrop et al. [51]	Both at baseline (R:-0.53; P:0.003) and at 6 weeks follow-up, copeptin was inversely correlated with eGFR (R:-0.56;P:0.002)	-No correlation between copeptin and ACR at baseline. -After 6 weeks a positive correlation was observed (r:0.44, P: 0.02)	28 patients with stage 3 CKD randomised to a hydration (to drink approximately 1 L more per day (n:17) than controls (n:11) for 6 weeks)	-In the hydration group, median copeptin decreased by 3.6 pmol/L, (P:0.005), while remaining stable among controls at 19 pmol/L (P:0.76).
Roussel et al. [52]	No data	No data	1.234 participants from the French general population with baseline co-peptin levels and followed for 9 years	Copeptin was associated with CKD according to KDIGO criterion: OR 3.03 (95% CI 1.21–7.57), P:0.02
Hu et al. [53]	Serum copeptin negatively related to GFR (R:-0.586, *P* < 0.001)	Co-peptin correlated with UAE (R:0.171, P:0.008)	120 T2DM patients	-Serum copeptin is an independent risk factor of decline in renal function in T2DM patients (OR:1.234, CI:1.003-1.456, p:0.012) -Sensitivity and specificty of the co-peptin in detection of GFR decline by roc analysis were 78.9% and 88.9% respectively
Nakajima et al. [54]	No correlation between urinary co-peptin and eGFR	No data	50 patients with ADPKD	Urinary copeptin/u-Cr was associated with total kidney volume and height-adjusted total kidney volume in ADPKD
Ponte et al. [11]	-In both men and women eGFR is negatively associated with copeptin	Subjects with pathologic 24-h ACR had higher copeptin levels compared to subjects with normal ACR (5.0 pmol/L [IQR, 3.2–8.7] vs. 3.9 pmol/L [2.7–5.8];P:0.001).	Population based study of 529 women and 481 men	-Subjects with simple cysts had higher copeptin levels compared to patients without cysts (4.8 pmol/L [IQR, 3.6–7.9] versus 3.8 pmol/L [2.6–5.7]; P:0.001) -The number of cysts in the kidneys is associated with copeptin -In adjusted models, copeptin remained its association with CKD (OR, 2.82; 95% CI, 1.45 to 5.50; P:0.002) and ACR (OR, 1.70; 95% CI, 1.08 to 2.68; P:0.02
Tasevska et al. [3]	No data	No data	Derived from the population based MDCS	After multivariate adjustment copeptin was independently associated with significantly greater annual decline of eGFR according to the MDRD and CKD-EPI formula
Engelbertz et al. [5]	eGFR is lower in patients with elevated copeptin compared to patients with normal copeptin (41.4 vs. 70.1, P: 0.001)	Proteinuria was present in 35.8% of patients with elevated copeptin, but only in 15.5% of patients with normal copeptin (*P* < 0.001)	301 patients (35 had no CKD and the others have various degress of CKD) with an angiographically diagnosed stenosis ≥50%	During 180 days of follow-up, Multivariate Cox regression analysis showed that copeptin was the sole predictor for mortality (HR: 5.317 (95% CI 1.653-17.098, *P* = 0.005)
Schiel et al. [55]	Positive correlation between copeptin and GFR both in patients with type 1 diabetes (R:0.86, P: 0.021) and in healty controls (R: 0.61, P: 0.034).	No correlation with albuminuria	80 patients with type 1 diabetes and 61 healthy controls	In type 1 diabetic patients multivariate analyses showed that only GFR was associated with copeptin (β: 0.23, P:0.032). No independent association in healty controls

*GFR* Glomerular Filtration Rate, *eGFR* estimated GFR, *mGFR* measured GFR, *CKD* Chronic kidney disease, *CI* Confidence Interval, *HR* Hazard Ratio, *MDRD* Modification of Diet in Renal Disease, *CKD-EPI Chronic Kidney Disease Epidemiology Collaboration, ACR* Albumin/creatinine ratio, *T2DM* Type 2 diabetes, *MDCS* Malmö Diet and Cancer Study, *HD* Hemodialysis, *LV* Left Ventricle, *ROC* Recieving operation Characterisitcs, *ADPKD* Autosomal dominant polycstic kidney disease, *IQR* interquartile range, *uae* Urinary Albumin Excretion, *CV* Cardiovascular, *RAAS* renin–angiotensin–aldosterone system, *RAASi* RAAS inhibition, *RRT* Renal replacement treatment, *TKV* Total Kidney Volume, *CIMT* Carotid Intima Media Thickness

**Table 2:** Studies regarding relationship of copeptin and hypertension

Study	Subjects	Main finding
Banasiuk et al, 2014	53 essential hypertensive adolesants and 31 normotensive adolesants (control group)	-Hypertensive patients had higher serum copeptin levels (median, 267 vs. 107.3 (*P* <0.01) -Copeptin is positively correlated with both 24-h systolic and diastolic BP and night time BP load -In multiple regression models copeptin is independently associated with, systolic BP, uric acid and body mass index
Uzun et al, 2015	-76 newly diagnosed, non-treated, hypertensive patients -36 patients were dippers and 40 were non-dippers	-The mean copeptin values were found to be significantly higher in the nondipper hypertensive group [1.66 (1.19–4.01) and 1.35 (1.12–2.09) IU/ml, respectively, *P* = 0.026]. -There were weak positive correlations between copeptin and 24-h daytime systolic (r: 0.350, *P* = 0.027) and diastolic BP (r:0.372, *P* = 0.018) -The correlations between copeptin and nocturnal systolic (r: 0.593, P < 0.0001) and diastolic BPs(r: 0.523, *P* = 0.001) are stronger compared to daytime values
Schoen et al, 2015	-prospective cohort study of 2012 healthy individuals between 25 and 41 years	-In multivariable linear regression models, log transformed copeptin was significantly associated with systolic and diastolic night-time BP levels among men but not among women. -Copeptin was strongly associated with an increased systolic and diastolic daytime and night-time BP variability -There was no relationship between copeptin and daytime BPs both in men and women -There is significant correlation between copeptin and nighttime BP among men but not among women
Mendes et al,2016	-140 patients with resistant hypertension (defined as supine office BP of at least 140 and/or 90 mmHg who received a 4-week standardized triple therapy regimen, including hydrochlorothiazide (12.5 mg/day), irbesartan (300 mg/day), and amlodipine (5 mg/day) and 26 patients with controlled hypertension -Resistant hypertensive patients were then randomized for 12 weeks of sequential nephron blockade (n:74) or sequential RAS blockade (n:66).	-Plasma copeptin concentrations was higher in resistant hypertension compared to controlled hypertension ([geometric mean 5.7 (confidence interval 95% 5.1–6.4) vs. 2.9 (2.3–3.9) fmol/ml, adjusted *P* < 0.0001). -At 12 weeks, plasma copeptin concentration in patients whose BP was controlled by sequential nephron blockade or sequential RAS blockade [6.8 (5.6–8.2) and 4.3 (3.0–5.9) fmol/ml, respectively) remained significantly higher than in patients with CBP at baseline (*P* < 0.0001 vs. both)
Schwerg et al, 2016	-40 resistant hypertensive patients (defined as ABPM (ambulatory blood pressure monitoring) > 135 mmHg over 24 h despite treatment with at least three antihypertensive drugs at the maximum tolerated doses including a diuretic) who underwent renal sympathetic denervation (RDN).	-The responder rate was 47.5% on 24 h ABPM. (defined as a drop in systolic ABPM 5 mmHg) -The mean systolic 24 h blood pressure dropped from 152 ± 10 mmHg to 147 ± 17 mmHg (p: .044) in the six month follow up. -Diastolic blood pressure values decreased from 83 ± 11 to 81 ± 15 mmHg in the entire group (p:0.26) -The mean baseline level of Copeptin was 7.4 pmol/l (interquartile range 3.7–11.6) for responders and 8.4 pmol/l (interquartile range 5.7–11–8) for non-responders (p:0.53). -Copeptin levels did not change over time after renal denervation.

*BP* Blood Pressure, *RAS* Renin–angiotensin system, *ABPM* Ambulatory Blood Pressure Monitoring, *RDN* Renal sympathetic denervation

## Discussion

In the current manuscript the relationship between copeptin, albuminuria and GFR was reviewed. After the review of manuscripts regarding these issues some findings were emerged such as:

i) Copeptin and GFR is usually negatively correlated
ii) Copeptin and albuminuria/proteinuria is positively correlated
iii) Copeptin and elevated BP were usually associated with each other

Many studies have shown that increased copeptin concentrations are linked to renal insufficiency and copeptin is negatively associated with estimated glomerular filtration rate (eGFR) [5, 8]. Why increased copeptin was associated with GFR and with CKD? The exact mechanisms regarding the relationship between copeptin, albuminuria and GFR are not known but two mechanisms were suggested. First, as copeptin is cleared by kidney excretion, copeptin levels would tend to increase as kidney function decreases. Second, in patients with lower kidney function, more copeptin is released, because the AVP system is activated due impaired urine concentrating capacity to maintain water homeostasis [9]. However, these ideas were challenged as it was shown that copeptin was not associated with GFR in healthy living kidney donors and copeptin levels did not change after donation despite a significant drop in kidney function after nephrectomy. These data suggest that GFR alone is not a principal determinant of copeptin [10]. Indeed, longitudinal studies in humans have shown that plasma copeptin levels increase before eGFR decreases [11].

In most studies copeptin was positively associated with urinary albumin/protein excretion [5] Population-based studies have shown copeptin to be strongly associated with microalbuminuria [12]. It was suggested that increased AVP might have albuminuric effect [11]. Indeed, V2 antagonists decrease proteinuria in animal models, one can hypothesize that albuminuria is somehow related to tubular V2Rs [13, 14]. Besides well-known antidiuretic effects at the collecting duct level, a V2-receptor agonist was shown to induce glomerular hyperfiltration and to increase UAE in normal rats [14, 15].

**Fig. 1:**
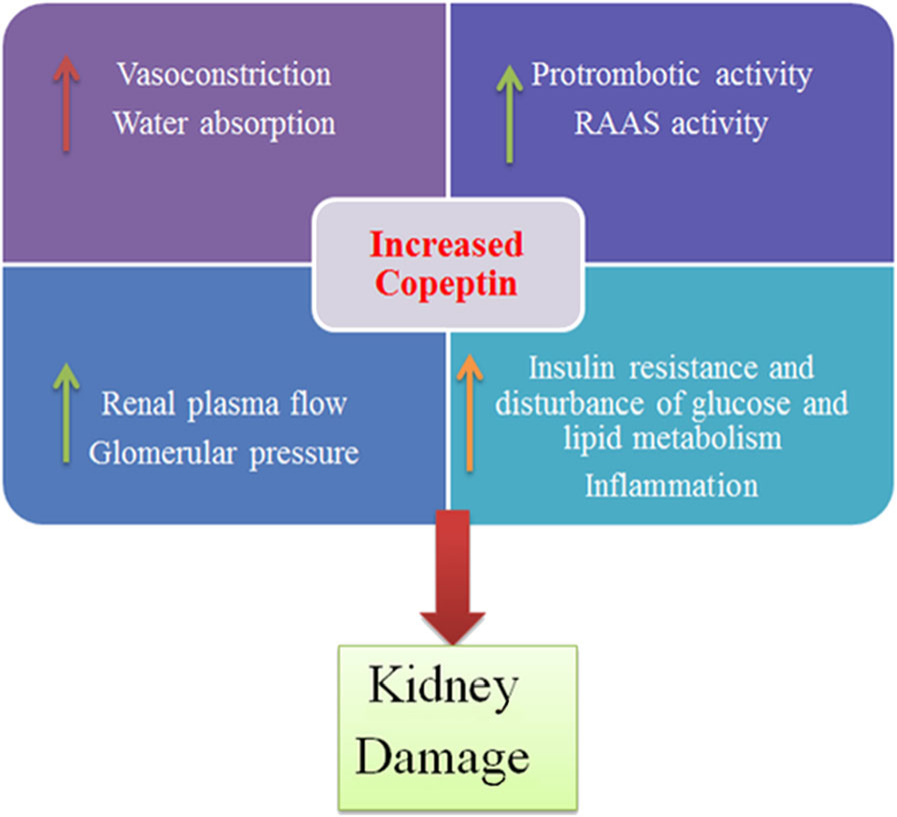
Potential mechanisms of increased copeptin with regard to worsening of kidney function. RAAS: Renin Angiotensin Aldosterone System

What are the mechanisms behind the adverse effects of copeptin on renal function? This question is not answered completely although some mechanisms were suggested (Fig. 1). In the following section these mechanisms are discussed.

Apart from classical stimulants for AVP secretion such as drop in blood pressure and hyperosmolarity, copeptin is also a marker of the body’s endocrine stress response, which is mediated through the hypothalamus–pituitary–adrenal system, and is activated in acute illness [16]. For example, copeptin levels spike in concert with cortisol and corticotropin-releasing hormone within hours of acute myocardial infarction onset [17]. It is unclear, however, whether copeptin is simply a marker of stress or illness, or if it plays a direct causative role in the pathophysiology of cardiovascular and chronic kidney disease [18].

Based on the studies and findings mentioned, one can speculate that elevation of AVP plays a role in the development of CKD, presumably through an effect on the V2R. Thus, increased water intake or pharmacological vasopressin blockade are interesting candidates for preventing the decline of eGFR and development of CKD. Water ingestion, which readily decreases circulating AVP/copeptin levels, may modify CKD progression [19], Studies in the 5/6 nephrectomized rat model suggested that increased water intake decreases circulating AVP levels and slows down the progression of kidney disease [20]. As suggested above increased GFR [15] and resulting proteinuria [14] causing and accelerated renal function decline [21] and tubulo-interstitial fibrosis [22]. These findings can be considered as adverse actions of increased copeptin on kidney function.

AVP also suggest influencing composition of the tubular fluid at the macula densa that influence tubuloglomerular feedback control of GFR, as well as an increase in intraglomerular pressure subsequent to afferent arteriole vasodilatation. Results obtained in rodent models of diabetes suggest that the underlying mechanism may be that AVP leads to hyperfiltration and then to albuminuria and glomerulosclerosis [23]. Indeed, it has been suggested that high AVP levels stimulate RAAS, resulting in vasoconstriction and consequently higher systemic and glomerular BP [24].

AVP has also other deterious actions. For example AVP play a role in glucose homeostasis, insulin resistance, and lipid and fat metabolism [25–27]. AVP has prothrombotic properties [28] and induces von Willebrand factor release from endothelial cells [29]. It also induces secretion of endothelin 1 and prostaglandin D2 from endothelial cells [30] which aggravates the diabetes-associated endothelial dysfunction and altered coagulation. Chronic inflammation may be other explanation. Several studies have shown that proinflammatory cytokines can activate VP secretion [31]. Therefore, inflammation could induce both VP secretion and accelerated decline in renal function.

The relationship between copeptin and hypertension is also worth to mention. Most of the studies have shown a positive association with copeptina and hypertension (Table 2) Recent evidence suggests that elevated blood pressure is associated with increased copeptin levels. For example, in hypertensive adolescents, copeptin levels were higher in normotensive adolesants. Not only office blood pressure but ambulatory blood pressures (both systolic and diastolic) were associated with copeptin levels [32–34]. In another recent study, the relationship between copeptin and resistant hypertension were investigated. Baseline plasma copeptin concentration was positively associated with male sex, plasma osmolality, BP, and negatively with glomerular filtration rate. It was higher in the resistant hypertension than in the controlled blood pressure group [geometric mean 5.7 (confidence interval 95% 5.1–6.4) vs. 2.9 (2.3–3.9) fmol/ml, adjusted *P*  <  0.0001) [35]. In fact older studies have already suggested that AVP may have a role in development of hypertension [36, 37]. However, Kawano et al. demonstrated that AVP did not play an important role in mild essential hypertension [38–40]. The lack of consensus on the role of vasopressin in essential hypertension may be the result of the fact that AVP is an unstable molecule both in vivo and ex vivo. In contrast to copeptin is stable molecule and it is considered to be a reliable and clinically useful surrogate marker for AVP. As suggested, copeptin has been associated with elevated blood pressure in various studies. Several lines of evidence suggest a role of copeptin in hypertension. One of the suggested mechanisms is the local tissue Renin Angiotensin Aldosterone System (RAAS) activation in supraoptic and paraventricular nuclei which stimulates the production and release of arginine vasopressin. Second mechanisms involve the vasoconstriction. This vasoconstriction is due to both direct effects on smooth muscle cells and by indirectly increasing renin secretion [32]. Third mechanism is the effect of copeptin on increased tubular sodium retention [41]. Thus copeptin may be common marker for essential hypertension and kidney disease.

The role of copeptin in renal denervation was also investigated [42]. Schwerg et al, investigated the change in copeptin levels in 40 resistant hypertensive patients after renal sympathetic denervation (RDN).- The responder rate was 47.5% on 24 h ABPM. which was defined as a drop in systolic ABPM 5 mmHg. The mean systolic 24 h blood pressure dropped from 152 ± 10 mmHg to 147 ± 17 mmHg (p :044) and diastolic blood pressure values decreased from 83 ± 11 to 81 ± 15 mmHg. (p:0.26 ) in the six month follow up. The mean baseline level of Copeptin was 7.4 pmol/l (interquartile range 3.7–11.6) for responders and 8.4 pmol/l (interquartile range 5.7–11–8) for non-responders (p:0.53). The authors concluded that copeptin levels did not change over time after renal denervation [42].

By the light of aforementioned data it is hypothetical that copeptin/avp play a role for the development and progression of CKD. Therefore, the blockage of copeptin/avp may be beneficial in halting development of CKD. However before doing that, full mechanisms need to be clarified. Phase studies should be planned regarding the efficiency of copeptin/avp blockage. Lastly, it needs to be tested whether high water intake will decrease the incidence of CKD by reducing copeptin/avp.

## Conclusion

In conclusion, copeptin is related with kidney function and hypertension and serve as a prognostic tool in these clinical conditions. Various mechanisms are thought to be responsible. More research is needed to highlight underlying mechanisms.
